# Herb-Drug Pharmacokinetic Interactions: Transport and Metabolism of Indinavir in the Presence of Selected Herbal Products

**DOI:** 10.3390/molecules201219838

**Published:** 2015-12-10

**Authors:** Carlemi Calitz, Chrisna Gouws, Joe Viljoen, Jan Steenekamp, Lubbe Wiesner, Efrem Abay, Josias Hamman

**Affiliations:** 1 Centre of Excellence for Pharmaceutical Sciences, North-West University, Private Bag X6001, Potchefstroom 2520, South Africa; 20743149@nwu.ac.za (C.C.); 11320036@nwu.ac.za (J.V.); Jan.Steenekamp@nwu.ac.za (J.S.); Sias.Hamman@nwu.ac.za (J.H.); 2 Department of Medicine, University of Cape Town, Observatory 7925, South Africa; lubbe.wiesner@uct.ac.za (L.W.); efrem.abay@uct.ac.za (E.A.)

**Keywords:** herb-drug interactions, efflux, P-glycoprotein, cytochrome P450, Caco-2, LS180, metabolism

## Abstract

Patients receiving anti-retroviral drug treatment are sometimes simultaneously taking herbal remedies, which may result in pharmacokinetic herb-drug interactions. This study aimed to determine if pharmacokinetic interactions exist between selected commercially available herbal products (*i.e.*, Linctagon Forte^®^, Viral Choice^®^ and Canova^®^) and indinavir in terms of *in vitro* transport and metabolism. Bi-directional transport of indinavir was evaluated across Caco-2 cell monolayers in the presence and absence of the selected herbal products and verapamil (positive control). Metabolism of indinavir was determined in LS180 cells in the presence and absence of the selected herbal products as well as ketoconazole (positive control). The secretory transport of indinavir increased in a concentration dependent way in the presence of Linctagon Forte^®^ and Viral Choice^®^ when compared to that of indinavir alone. Canova^®^ only slightly affected the efflux of indinavir compared to that of the control group. There was a pronounced inhibition of the metabolism of indinavir in LS180 cells over the entire concentration range for all the herbal products investigated in this study. These *in vitro* pharmacokinetic interactions indicate the selected herbal products may affect indinavir’s bioavailability, but the clinical significance needs to be confirmed with *in vivo* studies before final conclusions can be made.

## 1. Introduction

Human immunodeficiency virus (HIV) infection and acquired immune deficiency syndrome (AIDS) remains one of the biggest health problems that the world and more specifically, sub-Saharan Africa, is facing. Many HIV positive patients are also taking other drugs as well as herbal medicines concurrently with their anti-retroviral (ARV) drugs [1,2]. Herbal remedies usually comprise of a complex mixture of phytochemicals, each with the potential of exerting pharmacological effects but may potentially also cause pharmacokinetic or pharmacodynamic interactions with other drugs [3,4].

Pharmacokinetic interactions caused by herbal products involve induction and/or inhibition of intestinal efflux proteins as well as intestinal and hepatic metabolising enzymes [3]. Of the active efflux transporters involved in herb-drug interactions, P-glycoprotein (P-gp) is of special importance. P-gp is an ATP-binding cassette family transporter, which is highly expressed on the apical membrane of intestinal epithelial cells [5]. Its main physiological function is believed to provide protection against the onslaught of endogenous and exogenous compounds due to its ability to transport various structural dissimilar compounds out of the epithelial cells. This action of P-gp to extrude drug molecules from the epithelium back into the gastro-intestinal lumen may cause reduction in the absorption of a drug and consequently results in decreased oral bioavailability thereof [5]. The cytochrome P450 (CYP) enzyme family accounts for approximately 30% of the hepatic metabolism activity and 70% of the intestinal wall metabolism activity [3,6,7]. Various isoforms of the CYP enzyme family exist, however, the most abundant form namely CYP3A4 is responsible for the metabolism of up to 70% of all drugs. Inhibition of CYP3A4 may result in increased bioavailability of orally administered drugs with potentiation of adverse effects and possible toxicity, whereas induction thereof will increase metabolism of administered drugs resulting in reduced oral bioavailability and efficacy of drugs [3].

Linctagon Forte^®^ is a herbal product used to prevent and treat colds, flu and respiratory tract infections. It contains extracts of *Pelargonium sidoides* (Geraniaceae) as well as the natural occurring compounds quercetin and bromelain. *Pelargonium sidoides* is a traditional South African medicinal herb, which is commonly used as treatment for diarrhea, dysentery, colds and upper respiratory tract infections and tuberculosis (also available in the commercial herbal product Umckaloabo^®^) [8,9,10].

Viral Choice^®^ is a natural product containing a variety of minerals, vitamins, amino acids, trace elements, phytosterols as well as extracts of *Echinacea purpurea* (Asteraceae) and *Allium sativum* (Alliaceae) [11,12,13]. Common uses of *E. purpurea* includes treatment of the common cold, mainly due to its proposed immune modulation, treatment of influenza, bronchitis, inflammation and it also has anti-fungal effects [11,13]. *Allium sativum*, better known as garlic, contains high concentrates of thiosulfinates that are sulfur-containing compounds, specifically allicin, the active substance present in garlic [14]. The bulbous root of the garlic plant is medicinally used either fresh, dehydrated or in a steam distilled oil form as an immune system modulator, anti-microbial and anti-cancer agent, as well as anti-atherosclerotic and lipid lowering agent [14,15].

Canova^®^ is a homeopathic product composed of diluted extracts from *Aconitum napellus* (Ranunculaceae), *Arsenicum album* (arsenic trioxide), *Bryonia alba* (Curcubitaceae), *Apis mellifica* (Apidae), *Lycopodium clavatum* (Lycopodiaceae), *Pulsatilla nigricans* (Ranunculaceae), Asafoetida, *Rhus toxicodendrum*, Barita carbônica, *Ricinus communis* (Euphorbiaceae), Silicea, Calcarea carbônica, *Conium maculatum* (Apiaceae), *Veratrum album* (Liliaceae), *Carapichea ipecacuanha* (Rubiacea), *Lachesis muta* (Viperidae) and *Thuya occidentalis* (Cupresaceae). Canova^®^ is known as an immune response modifier directed at macrophages that results in an increase in their production together with the release of cytokine TNFα in cancer and AIDS patients [16,17].

Indinavir (Crixivan^®^) is an ARV drug (protease inhibitor) that is a substrate for both P-gp and CYP3A4. Pharmacokinetic interactions between indinavir and herbs (e.g., St Johns’ Wort, Milk thistle, Gingko, Ginseng, Echinacea and garlic) have been shown to decrease its bioavailability [18]. The current study investigated if *in vitro* pharmacokinetic interactions in terms of efflux and metabolism modulation exist between indinavir and three selected commercially available herbal products (*i.e.*, Canova^®^, Linctagon Forte^®^ and Viral Choice^®^) that are often used by patients for immune boosting effects, sometimes in conjunction with prescribed medication. Identifying possible pharmacokinetic interactions between the selected herbal products and indinavir will enable dissemination of this information to healthcare providers. They will then be able to inform patients of potential negative effects of simultaneous consumption of the specific herbal products with indinavir so that patients can avoid it.

## 2. Results and Discussion

### 2.1. In Vitro Transport Studies

#### 2.1.1. Bi-Directional Transport of Indinavir in the Presence of Linctagon Forte^®^

**Figure 1:**
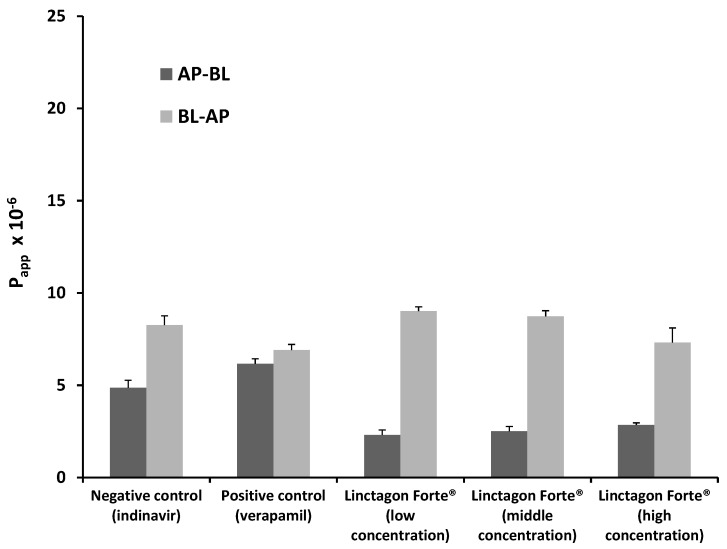
Bi-directional P_app_ values for indinavir in the presence of three concentrations of Linctagon Forte^®^ as well as for the negative control group (indinavir alone) and positive control group (indinavir with verapamil). *n = 3*, error bars indicate standard deviation.

The P_app_ values for indinavir in both directions for each of the Linctagon Forte^®^ test solutions as well as the control groups are presented in Figure 1. From this figure it can be seen that verapamil, a known inhibitor of P-gp, increased the uptake of indinavir in the AP-BL direction, whereas it decreased the efflux of indinavir in the BL-AP direction. In other words, the positive control group showed that the model is acceptable for testing modulation of efflux.

The overall transport of indinavir in the AP-BL direction decreased for all the concentrations of Linctagon Forte^®^ investigated in this study, while its transport was increased in the BL-AP direction. The medium concentration Linctagon Forte^®^ solution produced the most pronounced effect on indinavir efflux followed by the low and high concentrations, respectively. This “bell shape” dose dependent curve may implicate a saturation effect above a threshold concentration of Linctagon Forte^®^, but further investigations are needed to be conclusive in this regard. Furthermore, an increase in efflux may cause a reduction in the bioavailability of indinavir when simultaneously taken with Linctagon Forte^®^, but the clinical significance of this effect should be investigated by means of clinical trials.

Linctagon Forte^®^ is composed of *Pelargonium sidoides* extract, quercetin and bromelain. An inhibitory action of quercetin on P-gp activity has been shown previously by means of a rhodamine-123 transport study [19]. In another study by Borska *et al.* [20], the cell line EPG85-257RDB (RDB-cells) exposed to 12 µM quercetin showed a significant reduction in the expression of P-gp. Bromelain is absorbed by means of a self-enhanced paracellular route through the intercellular spaces [21,22]. It is thus unlikely that bromelain will have an effect on the P-gp transporters, since it is not taken up into the epithelial cells. Although no pharmacokinetic interaction due to modulation of P-gp is known for *P. sidoides* [23], the findings of this study suggest that the overall increase in efflux of indinavir (*i.e.*, a stimulation effect on efflux transporters such as P-gp) observed for Linctagon Forte^®^ may be attributed to modulation of the active transporters by phytoconstituents of the *P. sidoides* extract component.

The relatively high efflux ratio (ER) values (Table 1) for indinavir in the presence of Linctagon Forte^®^ compared to the control groups confirm its efflux stimulation effect.

**Table 1:** Efflux ratio (ER) values for indinavir in the presence of the selected herbal products and control groups.

Experimental Group	ER Value	
	Average (*n* = 3)	* SD
Indinavir alone (negative control)	1.70	0.133
Indinavir with verapamil (positive control)	1.12	<1 × 10^−3^
Indinavir with Linctagon Forte^®^ low concentration	4.63	0.334
Indinavir with Linctagon Forte^®^ medium concentration	5.70	0.379
Indinavir with Linctagon Forte^®^ high concentration	3.46	0.982
Indinavir with Viral Choice^®^ low concentration	3.95	0.391
Indinavir with Viral Choice^®^ medium concentration	3.41	0.286
Indinavir with Viral Choice^®^ high concentration	2.58	0.328
Indinavir with Canova^®^ low concentration	2.11	0.023
Indinavir with Canova^®^ medium concentration	1.83	0.180
Indinavir with Canova^®^ high concentration	1.67	0.120

* SD = standard deviation.

#### 2.1.2. Bi-Directional Transport of Indinavir in the Presence of Viral Choice^®^

**Figure 2:**
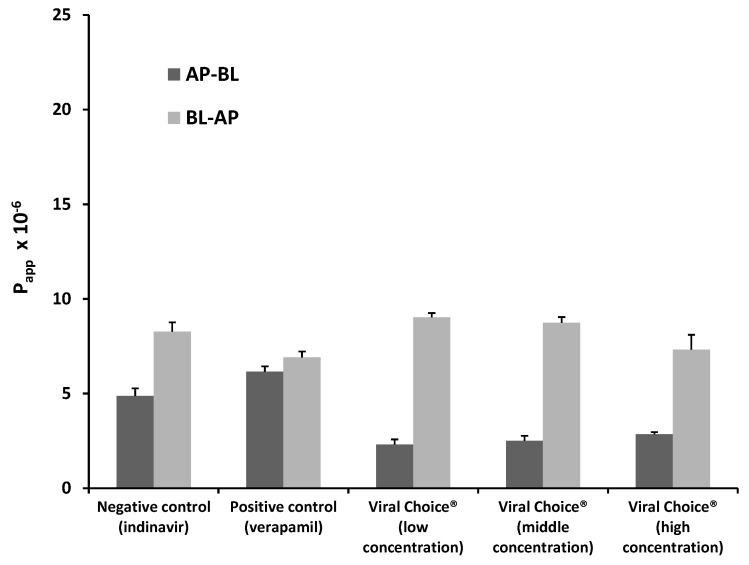
Bi-directional P_app_ values for indinavir in the presence of three concentrations of Viral Choice^®^ as well as for the negative control group (indinavir alone) and positive control group (indinavir with verapamil). *n = 3*, error bars indicate standard deviation.

The P_app_ values for indinavir in both directions for each of the Viral Choice^®^ test solutions as well as the control groups are presented in Figure 2. From this figure it is noticed that a decrease in the uptake of indinavir in the AP-BL direction occurred when combined with the Viral Choice^®^ solutions over the entire concentration range when compared to the negative control group (*i.e.*, indinavir alone). Efflux of indinavir in the BL-AP direction for the low and medium concentrations of Viral Choice^®^ increased to a certain extent. However, at the highest Viral Choice^®^ concentration, there was a slight decrease in the indinavir efflux from the BL-AP direction when compared to the negative control group, but not to the same extent as caused by verapamil in the positive control group. The trends observed for the P_app_ values of indinavir in the presence of Viral Choice^®^ (*i.e.*, increase of efflux at lower concentrations and decrease of efflux at higher concentrations) correspond exactly to those observed in a study by Hansen and Nilsen [12], which evaluated the effect of *Echinacea purpurea* extract (which is one of the main ingredients in Viral Choice^®^) on P-gp transport in Caco-2 monolayers.

#### 2.1.3. Bi-Directional Transport of Indinavir in the Presence of Canova^®^

**Figure 3:**
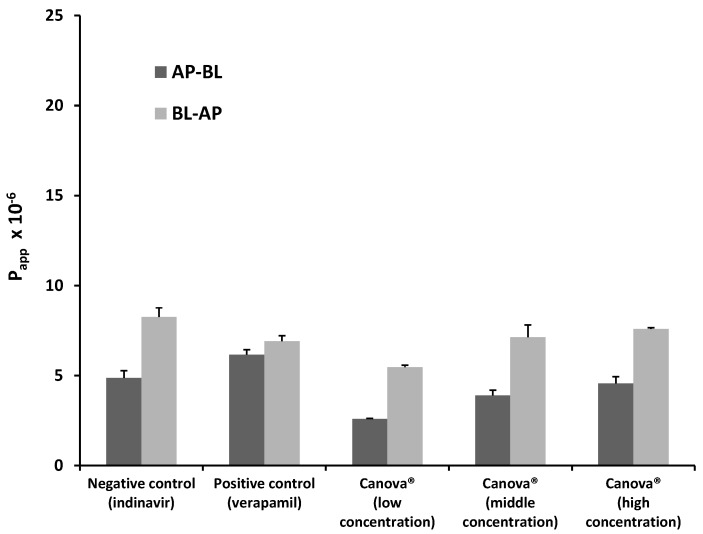
Bi-directional P_app_ values for indinavir in the presence of three concentrations of Canova^®^ as well as for the negative control group (indinavir alone) and positive control group (indinavir with verapamil). *n = 3*, error bars indicate standard deviation.

The P_app_ values for indinavir in both directions for each of the Canova^®^ test solutions as well as the control groups are presented in Figure 3. There was a concentration dependent increase in the percentage transport of indinavir (*i.e.*, AP-BL direction) in the presence of Canova^®^ as seen in Figure 3. The percentage uptake of indinavir in the presence of the lowest concentration Canova^®^ was lower than that of both the positive and negative control groups, whereas it was slightly elevated in the presence of the medium and high Canova^®^ concentrations in comparison to the negative control group (*i.e.*, indinavir alone). The efflux of indinavir (*i.e.*, transport in the BL-AP direction) was decreased by Canova^®^ at all the concentrations when compared to the negative control group. This effect is also reflected by the lower ER values of indinavir in the presence of Canova^®^ (Table 1) when compared to the other experimental groups where efflux was stimulated.

### 2.2. In Vitro Metabolism Studies

**Figure 4:**
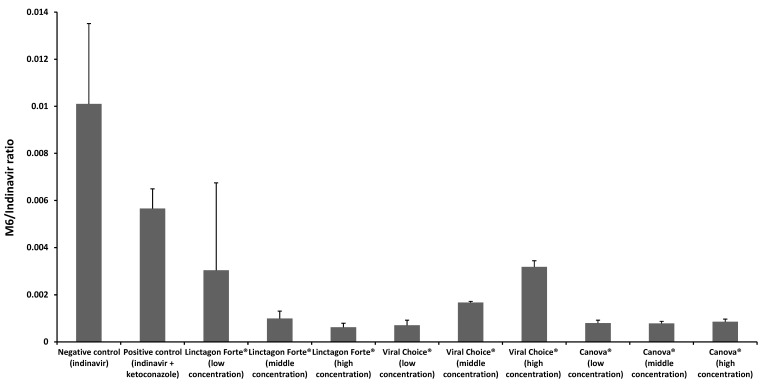
M6/indinavir ratios in the presence of Linctagon Forte^®^, Viral Choice^®^ and Canova^®^ with the negative control group (indinavir alone) and positive control group (indinavir with ketoconazole). *n =* 3, error bars indicate standard deviation.

Figure 4 provides a graphic representation of the results obtained from the *in vitro* metabolism study of indinavir in combination with ketokonazole (positive control) and the three selected herbal products (*i.e.*, Linctagon Forte^®^, Viral Choice^®^ and Canova^®^) at three concentrations (*i.e.*, low, medium and high), as well as indinavir alone (negative control group).

It is apparent from Figure 4 that there was a decrease in the concentration ratio of metabolite to parent drug (*i.e.*, M6:indinavir) in the positive control group compared to that of the negative control group, which indicated the usefulness of the LS180 cell culture model to investigate modulation of indinavir metabolism.

#### 2.2.1. Metabolism of Indinavir in the Presence of Linctagon Forte^®^

Linctagon Forte^®^ resulted in a concentration dependent inhibition of indinavir metabolism by LS180 cells. A 9 fold decrease in metabolism was observed with the highest concentration of Linctagon Forte^®^ when compared to the negative control group. This is consistent with a previous finding of at least one active constituent of Linctagon Forte^®^, namely quercetin. During an *in vivo* study on male Sprague-Dawley rats by Choi *et al.* [19], quercetin was capable of producing an inhibitory action on CYP3A4 in a concentration-dependent manner. The metabolism results therefore suggest a possible increase in the bioavailability of indinavir when given in combination with Linctagon Forte^®^, which is in contrast with the efflux stimulation effect that suggests a possible decrease in bioavailability of indinavir by this herbal product. Although these *in vitro* experiments identified potential pharmacokinetic interactions between Linctagon Forte^®^ and indinavir, *in vivo* investigations are needed to be conclusive regarding the final outcome with respect to the effect on the bioavailability of indinavir.

#### 2.2.2. Metabolism of Indinavir in the Presence of Viral Choice^®^

Viral Choice^®^ exhibited an inverse concentration dependent inhibition of indinavir metabolism in LS180 cells. An 8 fold decrease in inhibition was observed with the lowest concentration of Viral Choice^®^ when compared to the negative control group. It has previously been found that *E. purpurea*, one of the major active constituents of Viral Choice^®^, is capable of inhibiting CYP3A4. In an *in vitro* study by Budzinski *et al.* [24], extracts of the root and aerial portions of *E. purpurea* had an inhibitory effect on cDNA expressing CYP3A4. This was confirmed by an *in vivo* study conducted by Gorski *et al.* during which 400 mg of *E. purpurea* root was administered 4 times daily for 8 days [13]. They found that the clearance of caffeine and tolbutamide serving as *in vivo* probes for CYP1A2 and CYP2C9 metabolism was significantly reduced. Clearance of midazolam, the *in vivo* probe for hepatic CYP3A, was also significantly increased, which was consistent with preceding *in vitro* findings [13]. *Allium sativum* (Liliaceae), another active constituent of Viral Choice^®^, include high quantities of thiosulfinates that are sulphur containing compounds (e.g., allicin) [14]. However, varying effects on the CYP enzyme system have been reported, where some found inhibitory and some inducing effects on this enzyme system [15,25]. The results from this study suggest that the co-administration of Viral Choice^®^ may result in an increase in bioavailability of indinavir due to a reduction in the metabolism of indinavir.

#### 2.2.3. Metabolism of Indinavir in the Presence of Canova^®^

Canova^®^ markedly inhibited metabolism of indinavir by LS180 cells across the whole concentration range investigated. An overall 7 fold decrease in metabolism was observed for the entire concentration range of Canova^®^ when compared to the positive control. Tang *et al.* [26] investigated the involvement of various CYP isoenzymes in the metabolism of *Aconitum napellus* by means of human liver microsomes as well as recombinant CYP enzymes. It was determined that CYP3A4 and CYP3A5 were responsible for the metabolism of aconite whereas CYP2C8, 2C9, 2D6 and 2C19 played a slight role in the metabolism. Both studies suggest that co-administration of aconite together with other P-gp and CYP3A4/5 substrates may result in possible pharmacokinetic herb-drug interactions. In a study by Tam *et al.* [27], the effect of *L. clavatum* on the NADPH-dependent inhibition of CYP3A4 was examined. They found that *L. clavatum* significantly influenced both the NADPH and time dependent inhibition of CYP3A4 when combined with testosterone. This suggests that *L. clavatum*, similar to grapefruit juice, may possibly result in mechanism-based inactivation of CYP3A4. Asafoetida is the gum resin obtained from *Ferula asafoetida* (Apiacea) [28]. In a recent study by Al-Jenoobi *et al.* [29], the effect of asafoetida resin on the modulation of CYP2D6 and CYP3A4 was evaluated *in vitro* in human liver microsomes as well as in a clinical *in vivo* study. The *in vivo* study suggested that asafoetida significantly inhibits CYP3A4 activity with little to no inhibitory effect on CYP2D6 [29]. In another study regarding *Ricinus communis*, it was reported during an *in silico* study involving HerboChip^®^ followed by fluorometric enzyme inhibition studies that *R. communis* may have a significant effect on the inhibition of CYP3A4 [30]. The active constituents in *Carapichea ipecacuanha* are cephaeline and emetine amongst others. It was found that both these active constituents are substrates for CYP2D6 and CYP3A4. In an *in vitro* study involving human liver microsomes, both cephaeline and emetine, resulted in low inhibitory action against CYP2D6 as well as CYP3A4 [31]. The essential oil from *Thuya occidentalis* contains the active ingredient α-thujone which is a known substrate for CYP2A6 and CYP3A4. The latter suggests that co-administration with other CYP2A6 and CYP3A4 substrates may result in pharmacokinetic interactions [32]. From these published studies, together with the results obtained from the current metabolism inhibition study on Canova^®^, it is possible that these active constituents may have had a combined effect producing the pronounced overall inhibition of indinavir metabolism. This may potentially result in an increase of indinavir’s bioavailability, which will probably be potentiated by the efflux inhibition effect observed for Canova^®^. The results are surprising since homeopathic products are usually highly diluted in water, but even low concentrations of actives can be potent enough to exhibit an effect. In addition, the effects of excipients in the product cannot be excluded. This should be investigated further in future studies.

## 3. Experimental Section

### 3.1. Chemicals and Reagents

The three selected commercially available herbal products namely, Linctagon Forte^®^ tablets manufactured by Nativa, Viral Choice^®^ capsules manufactured by Winthrop Pharmaceuticals and Canova^®^ drops manufactured by Pharmachem pharmaceuticals, as well as Crixivan^®^ capsules (400 mg indinavir sulphate per capsule) manufactured by Merck Sharpe and Dohme Corp were purchased from a community pharmacy in Potchefstroom, South Africa. The composition for each selected herbal product investigated in this study is shown in Table 2, Table 3 and Table 4, respectively. The selected herbal products were chemically fingerprinted qualitatively by means of HPLC (results not shown). 

**Table 2:** Composition of Linctagon Forte^®^ tablets.

Active Constituent	Quantity per Tablet
*Pelargonium Sidoides*	250 mg
Quercetin	60 mg
Bromelain	40 mg

**Table 3:** Composition of Viral Choice^®^ capsules.

Active Constituent	Quantity per Capsule
Echinacea Extract	80 mg
Phytosterols (Plant sterols & sterolin)	25 mg
l-Arginine	10 mg
l-Methionine	33.3 mg
Absorption enzymes	5 mg
Garlic	50 mg
Vitamin A	333 RE
Vitamin B6	3 mg
Vitamin B12	4 µg
Folic acid	250 µg
Vitamin C	150 mg
Vitamin D	4 µg
Vitamin E	10 mg
Biotin	100 µg
Copper	0.33 mg
Iron	2 mg
Selenium	5 µg
Zinc	3 mg

**Table 4:** Composition of Canova^®^ drops.

Active Constituent	Quantity per mL
*Aconitum napellus*	DH 20 (0.06 mL)
*Apis mellifica*	DH 19 (0.06 mL)
*Arsenicum album*	DH 17 (0.06 mL)
Asafoetida	DH 20 (0.06 mL)
Barita carbônica	DH 20 (0.06 mL)
*Bryonia alba*	DH 14 (0.06 mL)
Calcarea carbônica	DH 20 (0.06 mL)
*Conium maculatum*	DH 16 (0.06 mL)
Ipecacaunha	DH 13 (0.06 mL)
*Lachesis muta*	DH 18 (0.06 mL)
*Lycopodium clavatum*	DH 20 (0.06 mL)
*Pulsatilla nigricans*	DH 13 (0.06 mL)
*Rhus toxicodendrum*	DH 17 (0.06 mL)
*Ricinus communis*	DH 14 (0.06 mL)
Silicea	DH 18 (0.06 mL)
*Thuya occidentalis*	DH 16 (0.06 mL)
*Veratrum album*	DH 20 (0.06 mL)

The prescribed dosage of each product, as directed by the package inserts, were ground with a mortar and pestle to a fine powder (where applicable) and dispersed in 100 mL distilled H_2_O. Each solution was stirred with a magnetic stirrer for 1 h. The solutions were filtered using a syringe filter (0.4 μm pore size) and evaluated by means of HPLC with the detector set at 270 nm. All other chemicals were of analytical grade and growth media were obtained from suppliers as indicated in the text of the methods below.

### 3.2. In Vitro Transport Studies

#### 3.2.1. Caco-2 Cell Culturing and Seeding on Transwell^®^ 6-Well Membrane Plates

The Caco-2 cell line was obtained from the European Collection of Cell Cultures (ECACC), (Sigma Aldrich, Johannesburg, South Africa). The cells were subsequently cultured in high-glucose DMEM (Separations, Randburg, South Africa) supplemented with 10% foetal bovine serum (The Scientific Group, Randburg, South Africa), 1% non-essential amino acids (NEAA) (Whitehead Scientific, Cape Town, South Africa), 1% penicillin/streptomycin (100 units penicillin/mL; 100 µg streptomycin/mL) (Separations), 2 mM l-glutamine (Whitehead Scientific) and 1% amphotericin B (250 µg/mL) (The Scientific Group). The Caco-2 cells were cultured at 37 °C with 5% carbon dioxide and 95% humidified air in a Galaxy 170R incubator (Eppendorf Company, Stevenage, UK). The growth medium was exchanged every second day.

Prior to the transport studies the Caco-2 cells were seeded onto Transwell^®^ 6-well membranes (Corning Costar^®^ Corporation, Tewksbury, MA, USA) with a pore diameter of 0.4 µm and surface area of 4.67 cm^2^. A cell suspension was obtained by means of trypsinisation with a Trypsin-Versene (EDTA) mixture (Whitehead Scientific). Cells in the cell suspension were counted by means of a haemocytometer after addition of Trypan blue (Sigma Aldrich) with a light microscope (Nikon Eclipse TS100/TS100F, Nikon Instruments, Tokyo, Japan). The cell suspension was then diluted to a concentration of 20,000 cells per mL, which were seeded onto the membrane filters.

Seeding of the cells onto Transwell^®^ filter membranes occurred under sterile conditions in a laminar flow hood by pipetting 2.5 mL of the final cell suspension into each apical chamber of the membrane filter plate wells. The Caco-2 cells were grown for 21 to 24 days on the membrane filters to produce intact epithelial cell monolayers whilst the growth medium was replaced every second day under sterile conditions. These Caco-2 cell monolayers were used in the bi-directional transport studies described below.

#### 3.2.2. Herbal Product Solution Preparations

To avoid application of unrealistic concentrations in the *in vitro* experiments, a concentration range for each of the selected herbal products were based on an extreme range of possible *in vivo* concentrations at different sites as described before [33]. Based on this method, the highest test solution concentration consisted of a prescribed dose (as outlined in the package insert) of each herbal product diluted to 500 mL, the medium concentration consisted of a dose of each herbal product diluted to 5 L and the lowest concentration consisted of a dose of each herbal product diluted to 50 L.

#### 3.2.3. *In Vitro* Bi-Directional Transport Studies

Prior to commencement of the transport experiments, the transepithelial electrical resistance (TEER) of each cell monolayer was measured with a Millcell ERS II meter (Millipore, Billerica, MA, USA) as an indication of the integrity thereof. A TEER reading higher than 250 Ω per cell monolayer (which is equivalent to 1167.5 Ω/cm^2^) was required before the transport studies could be conducted. At the end of the transport study, the TEER was measured again to confirm that the integrity of the cell monolayer was still intact after exposure of the cells to the test solutions.

For the transport studies in the apical to basolateral (AP-BL) direction (uptake), the growth medium was removed from the basolateral chamber of the Transwell^®^ membrane plate and replaced with 2.5 mL pre-heated DMEM buffered with HEPES (2-[4-(2-hydroxyethyl)piperazin-1-yl]ethanesulfonic acid) (The Scientific group) and incubated for 30 min at 37 °C. The growth medium was removed from the apical chamber and replaced with 2.5 mL of each of the test solutions (*i.e.*, each selected herbal product at three concentrations in the presence of 200 μM indinavir). Samples (200 µL) were withdrawn from the basolateral side at pre-determined time intervals of 20, 40, 60, 80, 100 and 120 min, which were each replaced with 200 µL of the pre-heated DMEM buffered with HEPES. Samples were analysed by means of high performance liquid chromatography (HPLC) to determine the indinavir concentration in the acceptor chamber over time.

For the transport studies in the basolateral to apical (BL-AP) direction (secretory transport), the growth medium was removed from the apical chamber and replaced with 2.5 mL pre-heated DMEM and incubated for 30 min at 37 °C. The growth media was removed from the basolateral chamber and replaced with 2.5 mL of each of the test solutions (*i.e.*, each selected herbal product at three concentrations in the presence of 200 μM indinavir). Samples (200 µL) were withdrawn from the apical side at pre-determined time intervals of 20, 40, 60, 80, 100 and 120 min, which were each replaced with 200 µL pre-heated DMEM. Samples were analysed by means of HPLC to determine the indinavir concentration in the acceptor chamber over time.

Indinavir alone was employed as a negative control, with verapamil (100 µM), a known P-gp inhibitor, in combination with indinavir as a positive control group. All experiments were performed in triplicate.

The apparent permeability coefficient (P_app_ × 10^−6^ cm/s) values for indinavir transport in each direction across the Caco-2 cell monolayers were calculated with equation 1 [4]. The P_app_ values (cm/s) represent the diffusion rate of the compound normalised for surface area of the cell monolayers and drug concentration on the donor side.

(1) $${\text{P}}_{\text{app}}=\frac{\text{dQ}}{\text{dt}}\left(\frac{1}{\text{A}\cdot {\text{C}}_{0}\cdot 60}\right)$$

where dQ/dt represents the increase in the amount of drug present in the acceptor compartment over time (slope of % transport curve), A is the effective surface area of the cell monolayer (cm^2^) that is exposed to the test solutions and C_0_ (µg/mL) is the initial drug concentration present in the donor chamber.

The efflux ratio (ER) value indicates any asymmetry in the directional transport of indinavir in combination with the various herbal products. The ER of indinavir transport was calculated by means of Equation (2) [4].

(2) $$\text{ER}=\frac{{\text{P}}_{\text{app}}\left(\text{BL}-\text{AP}\right)}{{\text{P}}_{\text{app}}\left(\text{AP }-\text{BL}\right)}$$

where AP-BL is the transport of indinavir in the apical to basolateral direction and BL-AP indicates the transport of indinavir in the basolateral to apical direction.

### 3.3. In Vitro Metabolism Studies

#### 3.3.1. LS180 Cell Culturing and Seeding out in 6-Well Plates for Metabolism Studies

The LS180 cell line was obtained from the European Collection of Cell Cultures (ECACC) (Sigma Aldrich). LS180 cell line was chosen as the *in vitro* model for the metabolism experiments in this study because it expresses the CYP3A4 enzyme [34,35,36]. The cells were subsequently cultured in high-glucose DMEM (Separations) supplemented with 10% foetal bovine serum (The Scientific Group), 1% non-essential amino acids (NEAA) (Whitehead Scientific), 1% penicillin/streptomycin (100 units penicillin/mL; 100 µg streptomycin/mL) (Separations), 2 mM l-glutamine (Whitehead Scientific) and 1% amphotericin B (250 µg/mL) (The Scientific Group). The LS180 cells were cultured at 37 °C with 5% carbon dioxide and 95% humidified air in a Galaxy 170R incubator (Eppendorf Company).

#### 3.3.2. Seeding of LS180 Cells onto 6-Well Plates

The LS180 cells were seeded onto 6-well plates (Corning Costar^®^ Corporation). A cell suspension was obtained by means of scraping the cells from the culturing flask; the cell suspension was extensively agitated with a pipette to ensure a homogeneous suspension consisting of single cells. Cells in the suspensions were counted using a haemocytometer after addition of Trypan blue (Sigma Aldrich) using a light microscope (Nikon Eclipse TS100/TS100F, Nikon Instruments). The cell suspension was diluted to a concentration of 250,000 cells per mL.

Seeding of the cells onto the 6-well plates occurred under sterile conditions in a laminar flow hood by pipetting 2 mL of the final cell suspension into each chamber of the 6-well plates. The seeded LS180 cells were left to adhere to the culture treated bottom surfaces of the wells and cultured for 24 h prior to commencement of the metabolism inhibition studies.

#### 3.3.3. Metabolism Inhibition Studies

The growth medium was removed from the LS180 cells in the 6-well plates and replaced with 2.5 mL test solution (*i.e.*, each selected herbal product at three different concentrations in the presence of indinavir). Ketoconazole (40 μM) was added to indinavir (200 μM) in the positive control group, whereas indinavir alone served as negative control group. The LS180 cells in the 6-well plates were incubated with each of the test and control solutions for 4 h at normal culturing conditions, after which the solutions were removed and the cells were washed with 1 mL PBS (Sigma Aldrich). The cells were harvested by gently scraping each well in order to detach all the cells and transferring them to labelled micro-centrifuge tubes, which were centrifuged in a Sigma Refrigerated Centrifuge 3-16KL (Wirsam Scientific, Johannesburg, South Africa) for 5 min at 300 g to ensure lyses of the cell membranes. After centrifugation, the supernatant was removed and the cell pellets were re-suspended in 200 µL ice-cold methanol, vortexed for 40 s and kept at −80 °C until dried. Before drying, the cell suspension was thawed and centrifuged at 10,000 *g* for 10 min to remove proteins and cell debris. The supernatant was removed and placed in 2.5 mL micro-centrifuge tubes, which were then dried under nitrogen. Prior to LC-MS/MS analysis, the samples were reconstituted in 100 µL of 0.1% formic acid in water and vortex mixed for 1 min. All experiments were performed in triplicate.

The ratio of the concentration of the M6 metabolite (*N*-oxidation of aromatic nitrogen) of indinavir (*i.e.*, (2*R*)-1-[(2*S*,4*R*)-4-benzyl-2-hydroxy-4-{[(1*R*,2*S*)-2-hydroxy-2,3-dihydro-1*H*-inden-1-yl]-*C*-hydroxycarbonimidoyl}butyl]-*N*-*tert*-butylpiperazine-2-carboximidic acid) to that of parent indinavir concentration was calculated to indicate any modulation of indinavir metabolism in the LS180 cells by the herbal products. During inhibition of enzymatic activity, there should be a decline in the concentration of the metabolite in relation to the parent drug.

### 3.4. Chromatographic Analysis

#### 3.4.1. High Performance Liquid Chromatography Method

An HP1100n chromatograph equipped with a UV detector and Chemstation Rev .A.10.01 Agilent^®^ Technologies data acquisition and analysis software (Hewlett-Packard, Palo Alto, CA, USA) was used for both the fingerprinting of the herbal products and for the analysis of indinavir concentration in the transport samples.

The HPLC analysis was performed on a Venusil XBP C18(2) column, 4.6 × 150 mm, 5 µm, 100 Å (Agela Technologies, Wilmington, DE, USA) using acetonitrile adjusted to pH 7 with 0.1% ammonium formate (NH_4_HCO_2_) as the mobile phase in isocratic elution mode with a flow rate of 1 mL/min and an injection volume of 100 µL. The UV detector was set at 210 nm.

The following data were obtained during the validation of the HPLC method for indinavir: the graph where indinavir peak areas were plotted as a function of concentration over a concentration range of 0–24.93 µg/mL exhibited a linear regression (*R*^2^) value of 0.999. The system performance and repeatability proved well with relative standard deviation (RSD) values of 0.30% for peak area and 0.182% for retention time. Indinavir was stable in the sample solution over a period of 24 h. The method was able to tolerate small changes in the chromatographic conditions and performed well under normal use. It was concluded that the method was suitable to analyze indinavir in the transport samples.

#### 3.4.2. Liquid Chromatography Linked to Mass Spectroscopy (LC-MS/MS)

Isocratic liquid chromatography was performed on an SB-Phenyl (2.1 × 100 mm, 1.8 µm) column using an Agilent 1100 series HPLC instrument. The mobile phase consisted of a mixture of phase A and B (40:60 *v*/*v*), where phase A was 0.1% *v*/*v* formic acid in water and phase B was 0.1% *v*/*v* formic acid in acetonitrile. The mobile phase was delivered at a constant flow rate of a 180 µL/min and the injection volume was 5 µL, while the column was kept in a compartment at 40 °C.

Detection of indinavir and M6 (indinavir metabolite) was performed on an AB Sciex API 3200 mass spectrometer (ESI in the positive ion mode) and the settings on the apparatus are summarized in Table 5 and Table 6. The mass spectrometer was operated at unit resolution in the multiple reaction monitoring (MRM) mode, monitoring the transition of the protonated molecular ions at *m*/*z* 614.4 and 523.4 to the product ions at *m*/*z* 421.3 and 273.1 for indinavir and M6.

The following data were obtained during the validation of the LC-MS/MS method for indinavir: the accuracies (% Nom) for indinavir and M6 were between 87% and 113% at low, medium and high quality control (QC) levels during sample analysis. The percentage coefficient of variation (% CV) for indinavir and M6 during sample analysis was less than 13 at low, medium and high QC levels.

**Table 5:** Ionization source setting.

Electro Spray Ionisation Settings	Value
Nebulizer gas (Gas 1) (arbitrary unit)	30
Turbo gas (Gas 2) (arbitrary unit)	40
CUR (curtain gas) (arbitrary unit)	15
CAD (collision gas) (arbitrary unit)	3
TEM (source temperature) (°C)	500
IS (Ion Spray Voltage) (V)	3500

**Table 6:** MS/MS detector setting.

MS/MS Settings	Indinavir	M6
Protonated molecular mass (*m*/*z*)	614	523
Product ion molecular mass (*m*/*z*)	421	273
Dwell time (ms)	150	150
DP (declustering potential) (V)	45	40
EP (entrance potential) (V)	12	9
CEP(collision cell entrance potential) (V)	35	40
CE (collision energy) (eV)	50	45
CXP (collision cell exit potential) (V)	11	11
Scan Type	MRM	MRM
Polarity	Positive	Positive
Pause time	5ms	5ms

## 4. Conclusions

The selected herbal products (*i.e.*, Linctagon Forte^®^, Viral Choice^®^ and Canova^®^) showed *in vitro* modulation effects on both the efflux and metabolism of indinavir to different extents. Linctagon Forte^®^ and Viral Choice^®^ exhibited efflux stimulation, but metabolism inhibition of indinavir. Canova^®^ exhibited inhibition of both efflux and metabolism of indinavir. The latter may therefore increase the bioavailability of indinavir after simultaneous oral administration, however, *in vivo* pharmacokinetic studies are needed to be conclusive in terms of any significant bioavailability changes of indinavir by any of these herbal products.
